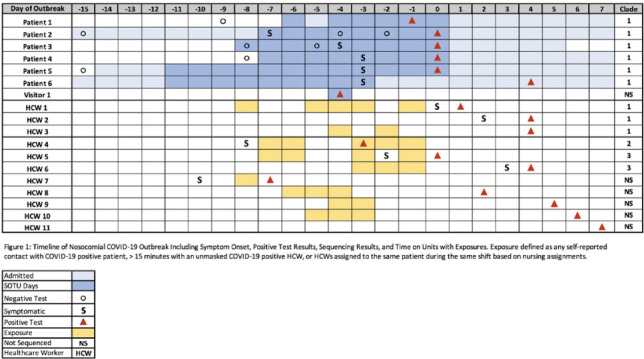# Nosocomial outbreak of δ (delta) variant SARS-CoV-2 on a liver transplant unit: A complex epidemiologic and genomic investigation

**DOI:** 10.1017/ash.2022.214

**Published:** 2022-05-16

**Authors:** Jonathan Ryder, Trevor Van Schooneveld, Baha Abdalhamid, Macy Wood, Richard Starlin, Gayle Gillett, Teresa Balfour, Libby Pflueger, Mark Rupp

## Abstract

**Background:** In late September 2021, a cluster of patients with nosocomial COVID-19 was identified on a liver transplant unit at University of Nebraska Medical Center. **Methods:** The outbreak investigation included contact tracing via patient chart and employee health record reviews and serial prevalence testing for SARS-CoV-2 among potentially exposed patients and healthcare workers (HCWs). Routine admission and preprocedural screening for SARS-CoV-2 was performed, and involved patients had negative admission screening results with positive SARS-CoV-2 tests >5 days from admission. Mitigation strategies involved reinforcement of patient care and visitation procedures. Whole-genome sequencing of positive SARS-CoV-2 specimens was conducted. **Results:** The potential outbreak cluster included 6 patients in the same quadrant of the liver transplant unit, 1 visitor, and 11 healthcare workers (Fig. 1). Moreover, 4 patients had severe liver disease, including 2 with liver transplants. All HCWs and half of the patients had received 2 doses of mRNA vaccine, albeit >5 months from their second vaccination. Whole-genome sequencing confirmed patients 1–6 and HCWs 1–3 had related transmission of COVID-19. However, infections in HCWs 4–6, who worked in a transplant-related office setting without patient contact, were due to 2 separate introductions of SARS-CoV-2 unrelated to the hospital outbreak. Sequencing could not be performed on HCWs 7–11 due to low viral concentration in the original specimens or unavailable specimen. The SARS-CoV-2 δ (delta) variant (B.1.617.2) was identified in all sequenced samples. HCWs 8–10 were asymptomatic and had had contact with each other and had been involved with an intubation without proper PPE for SARS-CoV-2 on patient 6. HCW 8 had had contact with all 6 patients and HCW 9 had had contact with 5 patients. A clear index case could not be identified; however, we suspect that the index case was either visitor 1, who tested positive during patient 2’s admission, or an asymptomatic healthcare worker (HCWs 8–10). **Conclusions:** We identified a nosocomial outbreak of the SARS-CoV-2 δ (delta) variant in a solid-organ transplant unit including patients, a visitor, and vaccinated healthcare workers with multiple introductions of the virus. Further transmission was not detected after enhanced infection control measures were introduced, including universal masking and eye protection, closing patient doors, and enforcement of visitor masking policy. We describe the difficulties tracing SARS-CoV-2 transmission in the hospital setting, even with advanced sequencing techniques. This outbreak highlights the importance of booster vaccination and strict infection control practices, especially in the setting of the δ (delta) variant.

**Funding:** None

**Disclosures:** None

**Figure:**